# Incidence of metabolic syndrome in patients with unilateral or bilateral staghorn renal stones and its impact on percutaneous nephrolithotomy outcomes

**DOI:** 10.1186/s12894-024-01526-4

**Published:** 2024-07-09

**Authors:** Zhonghua Shen, Linguo Xie, Di Luo, Haijie Xie, Hongyang Chen, Chunyu Liu

**Affiliations:** Department of Urology, Tianjin Institute of Urology, The Second Hospital of Tianjin Medical University, 23 Pingjiang Road, Hexi District, Tianjin, 300000 China

**Keywords:** Metabolic syndrome, Staghorn calculi, Percutaneous nephrolithotomy, Complication

## Abstract

**Background:**

To evaluate the incidence of metabolic syndrome (MetS) in patients with unilateral and bilateral staghorn calculi (SC) and evaluate the impact on the outcome of percutaneous nephrolithotomy (PCNL).

**Methods:**

The clinical data of patients who underwent PCNL for the treatment of SC between 2019 and 2022 were retrospectively reviewed. SC was divided into unilateral and bilateral. The incidence of MetS was compared between the patients with unilateral SC and the patients with bilateral SC, and the impact on the outcome of PCNL was assessed.

**Results:**

A total of 1778 patients underwent PCNL between 2019 and 2022. After screening computed tomography, 379 patients were confirmed to have SC, finally, leaving 310 patients with follow-up and complete data to be included in the study. Eighty-four had bilateral SC and 226 had unilateral SC. The patients with bilateral SC had a significantly higher body mass index and higher rates of complete staghorn stones and metabolic syndrome. Higher body mass index, hypertension, diabetes mellitus, hyperlipidaemia, and MetS were present in 62.58%, 44.84%, 21.94%, 60.65% and 27.42% of all patients, respectively. The number of MetS components remained significantly associated with bilateral SC. Specifically, when the number of MetS components increases from 0 to 3–4, the likelihood of developing bilateral staghorn calculi increases by 21.967 times. Eighty-five patients with MetS( +) had a higher rate of overall complications (number (N)(%), 29 (34.12) vs.33 (14.46), *P* < 0.001) and a comparable stone-free rate to 225 MetS(-) patients. Multivariable analysis confirmed that hyperlipidaemia (*P* = 0.044, odds ratio [OR] = 1.991, 95% confidence interval [CI] 1.020–3.888) and MetS (*P* = 0.005, OR = 2.427, 95% CI 1.316–4.477) were independent risk factors for overall complications.

**Conclusions:**

MetS is correlated with the formation of bilateral SC and is the main predictor for complications of PCNL especially for low-grade complications (I-II).

## Background

Kidney calculi are common health problem worldwide, with an incidence ranging from 5.9% to 9.1% [1, 2]. Staghorn renal calculi are one of the most complex types of upper urinary calculi. They are defined as large stones that occupy the renal pelvis involving two or more renal calices, and percutaneous nephrolithotomy (PCNL) is the recommended treatment according to major guidelines [3, 4]. PCNL remains a major challenge for urological surgeons because of the potential high risk of postoperative complications [5].

The formation of urinary calculi is closely related to an abnormal metabolism in the body. Metabolic syndrome (MetS) is a pathological state in which multiple metabolic disorders exist simultaneously in the body, mainly including central obesity, hypertension, abnormal glucose metabolism, and abnormal lipid metabolism [6]. Previous studies have shown a linear correlation between urinary calcium and body mass index (BMI) [7]. The supersaturation of urinary calcium was significantly higher in patients with type 2 diabetes [8]. In terms of blood lipids, it has been reported that high triglycerides are significantly associated with low urine pH, high oxaluria and high uric aciduria [9]. Patients with hypertension are more likely to have lower urinary pH and lower urinary citrate excretion [10]. A recent meta-analysis showed that MetS was associated with an increased risk of developing nephrolithiasis, and a significant linear association between MetS components and nephrolithiasis was revealed [11]. Our previous study found that MetS is an independent risk factor for severe urolithiasis [12].

Few studies have discussed the relationship between MetS and staghorn renal calculi. A previous study showed that metabolic disorders were found in nearly 70% of patients with staghorn renal calculi, especially patients with bilateral stones [13]. Another study showed that blood and urine metabolic factors were correlated with staghorn renal stones, especially bilateral staghorn stones [14]. A cross-sectional study of 10,281 participants with bilateral renal stones showed that high proportions of metabolic components (blood pressure and serum glucose) were associated with a higher risk of chronic kidney disease and higher levels of kidney tubular injury markers [15]. However, little is known about MetS and the component differences between unilateral and bilateral staghorn renal stones.

Few studies have been conducted assessing the relationship between metabolic syndrome and surgical complications associated with urinary calculi. The first initial quantified study found that MetS patients have an increased risk of postoperative myocardial infarction following PCNL [16]. A retrospective cohort study showed that the number of MetS components, as a central predictor, was suitable for determining the severity of MetS and the risks associated with PCNL for staghorn renal calculi [17]. However, there is little evidence to support this view. Considering the above problems, this retrospective study was conducted to evaluate the incidence of MetS in patients with unilateral and bilateral staghorn renal stones and to compare its impact on the outcome of PCNL.

## Methods

### Study design

A total of 1778 patients underwent PCNL between 2019 and 2022. After screening with computed tomography, 379 patients were confirmed to have staghorn stones, and patients without follow-up data or with incomplete data were excluded. A total of 310 patients with staghorn calculi who were treated at the Second Hospital of Tianjin Medical University from 2019 to 2022 were included in the study.

### Data collection

We analyzed the data of the patients who underwent PCNL. The preoperative factors that were studied included age, sex, weight, and comorbidities such as diabetes, hypertension, and hyperlipidaemia. All blood and urine samples were collected as previously described [12], and serum calcium, magnesium, potassium, uric acid, creatinine, triglycerides (TG), high-density lipoprotein cholesterol (HDL-C) and urine culture were included. Stone size, burden, CT value and sides were evaluated by imaging examinations, including urological ultrasound and renal computer tomography, which were performed for all patients before starting the procedure. Intraoperative factors included operative time and intraoperative complications. Operative time was defined from the time of ureteroscopy to the fixation of the nephrostomy tube. Stone-free status for stones < 3 mm (evaluated from renal computer tomography performed on the third postoperative day), incidence of postoperative complications, and hospitalization days were also recorded. Haemoglobin deficit was used to evaluate blood loss. The modified Clavien system was used for grading complications following PCNL [18]. For bilateral staghorn stones, only the intraoperative and postoperative conditions of one side were recorded (specifically, the side treated with the first PCNL). The preoperative CT value measurement for patients with stones is obtained from three planes, and the average value is calculated. For patients with bilateral stones, the average CT value is derived from both sides.

Staghorn stones were defined as large stones that occupy the renal pelvis involving two or more renal calices. Complete staghorn stones were defined as those occupying more than 80% of the collecting system. Staghorn stones those occupying no more than 80% of the collecting system were defined as partial staghorn stones.

The MetS diagnostic criteria were recommended by the Diabetes Branch of the Chinese Medical Association [6]. The following four criteria must meet at least three: (1) BMI ≥ 25.0 kg/m^2^; (2) fasting plasma glucose ≥ 6.0 mmol/L or been diagnosed with diabetes; (3) hypertensive (systemic blood pressure ≥ 140 mmHg or diastolic blood pressure ≥ 90 mmHg, or been diagnosed with hypertension and treated); (4) TG level ≥ 1.62 mmol/L or blood HDL-C level < 1.07 mmol/L, or been diagnosed with dyslipidemia and treated. BMI, TG, HDL-C, and the presence or absence of diabetes, hypertension, and hyperlipidaemia were recorded after admission and preoperatively. The number of MetS components was recorded to assess the risk of complications.

PCNL was performed under general anaesthesia. In the lithotomy position, a 5-French(Fr) ureteral catheter was inserted into the ureter via ureteroscopy. Then, the patient was repositioned to the prone position. Under ultrasound guidance, an 18-gauge needle, and a J-tipped guide wire was placed into the collecting system for percutaneous access. Tract dilatation was accomplished with an 16Fr, 18 Fr or 24Fr fascial dilator. Two-step expansion method was used to establish the 18Fr and 24Fr tract. The first step was to expand through the guide wire by the 8-16Fr fascial dilator (LAKH Medical Instrument), and then we inserted a ureteroscope to observe the tract and renal pelvis and made corresponding adjustments. 18Fr fascial dilator(LAKH Medical Instrument) was used to complete a 18Fr tract. For 24Fr tract a telescopic metal dilator (Richard Wolf) was inserted to dilate the working access from 15 to 21F, followed by the introduction of a16-cm-long, 24F metal working sheath (Richard Wolf) as a standard percutaneous tract. Stones were fragmented with high-power holmium laser then flushed out of the renal pelvis for 16Fr and 18 Fr tract; Stones were dusted with ultrasonic lithotripsy system(EMS Electro Medical Systems) for 24Fr tract. At the end of the procedure, a 6-Fr ureteric catheter was inserted, and a 16/20-Fr nephrostomy tube was fixed. Then, one or more supplementary percutaneous access tracts were added as required to achieve a satisfactory result. If there was no fever, bleeding and residual stone at 3–4 days after the operation, the 16/20-Fr nephrostomy will be removed. While the 6-Fr ureteric catheter was removed 4 weeks later before evaluated with KUB. All methods were carried out in accordance with relevant guidelines and regulations.

For the treatment of bilateral staghorn kidney stones, patients underwent staged procedures at different times, with initial treatment focusing on unilateral kidney stones. Treatment priority is based on the patient's symptoms and infection status, with the infected and severely hydronephrotic kidney being addressed first. However, if the patient has a functional solitary kidney, priority is given to the side with better kidney function. Typically, a second surgery is performed 2–4 weeks later to treat residual or contralateral stones.

### Statistical analysis

The data were stored and analysed using SPSS (20.0). We used the chi-square test for qualitative variables. Normally distributed continuous variables were expressed as mean ± standard deviation(Student's t-test), non normally distributed continuous variables were expressed as mean (interquartile range, nonparametric test). Univariable and multivariable logistic regression analyses were employed to evaluate the association between metabolic syndrome and bilateral staghorn renal stones, as well as overall complications. When conducting regression analysis for continuous variables, the Receiver Operating Characteristic (ROC) curve is plotted, and the value with the highest Youden index is selected as the cutoff value. A *P* value < 0.05 was accepted as statistically significant.

## Results

A total of 1778 patients underwent PCNL between 2019 and 2022. After screening computed tomography, 379 patients were confirmed to have staghorn calculi, but patients without follow-up data or with incomplete data were excluded, leaving 310 patients with staghorn calculi to be included in the study. Eighty-four patients had bilateral staghorn renal stones, and 226 had unilateral staghorn renal stones. The mean patient age, serum calcium, serum magnesium, serum kalium, serum uric acid, serum creatinine and CT value were comparable in the two groups. The ratio of male to female, hydronephrosis, and positive urine culture were no difference in the two groups. Higher proportions of patients with a BMI ≥ 25 kg/m^2^(65(77.38%) vs. 129(57.08%), *P* = 0.001), hypertension (48(57.14%) vs. 91(40.27%), *P* = 0.008), diabetes mellitus (29(34.52%) vs. 39(17.26%), *P* = 0.001), hyperlipidaemia (62(73.81%) vs. 120(53.10%), *P* = 0.001), and MetS (38(45.24%) vs. 47(20.80%), *P* < 0.001) were in bilateral staghorn renal stones group. More patients had complete staghorn calculi (32 (38.10%) vs. 54 (23.89%), *P* = 0.013) (Table 1).

**Table 1:** Preoperative characteristics of the patients according to the state of straghorn stone

Variable	Unilateral	Bilateral	*P* value
Number of patients	226	84	-
Mean (SD)			
Age, years	55.34(11.42)	56.05(11.08)	0.239
Body mass index, kg/m2	25.95(4.01)	27.19(3.59)	0.013
Serum uric acid(mmol/L)	361.12(110.85)	370.06(92.24)	0.511
CT value(HU)	1073.38(343.97)	1027.48(342.94)	0.297
Mean(Min–Max)			
Serum magnesium (mmol/L)	0.90(0.38–1.48)	0.87(0.69–1.06)	0.340
Serum kalium (mmol/L)	3.93(3.0–5.0)	3.89(2.7–4.9)	0.964
Serum creatinine (umol/L)	84.59(43.6–502.5)	87.07(38.6–343.5)	0.135
Serum calcium (mmol/L)	2.33(1.19–3.08)	2.33(1.05–2.7)	0.723
N (%)			
Sex			
Males	115(50.88)	51(60.71)	0.124
Females	111(49.12)	33(39.29)	
Hydronephrosis			
No	47(20.80)	26(30.95)	0.116
Mild to moderate	165(73.01)	53(63.10)	
Marked	14(6.20)	5(5.95)	
Positive urine culture	69(30.61)	18(26.89)	0.114
Stones classification			
Partial staghorn	172(76.11)	52(61.90)	0.013
Complete staghorn	54(23.89)	32(38.10)	
Comorbidities			
Higher body mass index	129(57.08)	65(77.38)	0.001
Hypertension	91(40.27)	48(57.14)	0.008
Diabetes mellitus	39(17.26)	29(34.52)	0.001
Hyperlipidemia	120(53.10)	62(73.81)	0.001
Metabolic syndrome	47(20.80)	38(45.24)	< 0.001

*SD* standard deviation, *N* number, *CT* computed tomography, *HU* hounsefield units

A higher BMI, hypertension, diabetes mellitus, hyperlipidaemia, and MetS were observed in 62.58%(with 65 in bilateral SC group and 129 in unilateral SC group), 44.84%(with 48 in bilateral SC group and 91 in unilateral SC group), 21.94%(with 29 in bilateral SC group and 39 in unilateral SC group), 60.65%(with 62 in bilateral SC group and 120 in unilateral SC group) and 27.42%(with 38 in bilateral SC group and 47 in unilateral SC group) of all patients, respectively (Table 1).

To observe the effect of the number of metabolic syndrome components on the formation of bilateral SC, the number of MetS components were divided into 0–4. We found that as the number of metabolic syndrome components increases, the risk of bilateral staghorn calculi increases ( For SC, when the number of MetS components was zero, only a 3.33% chance of bilateral staghorn stones occurring; But when the number of MetS components roes to four, the chance of bilateral staghorn stones increases to 50%) (Table 2).

**Table 2:** The effect of the number of metabolic syndrome components on staghorn stone

Number of MetS components	Unilateral staghorn stones(226)	Bilateral staghorn stones(84)	*P* value
0	29(96.67%)	1(3.33%)	0.001
1	70(86.42%)	11(13.58%)	0.001
2	80(70.18%)	34(29.82%)	0.244
3	38(56.71%)	29(43.28%)	0.001
4	9(50.00%)	9(50.00%)	0.028

*MetS* Metabolic syndrome

To conduct regression analysis, for continuous variables, the ROC curve is plotted, and the value with the highest Youden index is selected as the cutoff value. For age, the cutoff value was 57 yrs; For uric acid, magnesium, kalium, calcium the cutoff value were 284.85, 0.79, 4.05, and 2.35 mmol/L; For creatinine, the cutoff value was 85.8 ummol/L. Results from univariate logistic regression analysis showed that variables such as serum creatinine and the number of MetS components were associated with the formation of bilateral staghorn calculi. However, after multivariate logistic regression analysis, it was found that only the number of MetS components remained significantly associated with bilateral SC. Specifically, when the number of MetS components increases from 0 to 3–4(Mets( +)), the likelihood of developing bilateral staghorn calculi increases by 21.967 times (Table 3).

**Table 3:** Univariate and multivariate analysis predictive of factors for bilateral staghorn stone

Variable	Univariate analysis		Multivariate analysis	
	OR(95%CI)	*P* value	OR(95%CI)	*P* value
Age: ≤ 57 yrs, > 57 yrs	0.640(0.387–1.059)	0.082		
Sex: male,female	1.492(0.896–2.483)	0.124		
Urine culture: negative, positive	0.621(0.343–1.123)	0.115		
Uric acid(mmol/L): ≤ 284.85, > 284.85	1.890(0.993–3.599)	0.053		
Magnesium (mmol/L): ≤ 0.79, > 0.79	1.546(0.789–3.028)	0.204		
Kalium (mmol/L): ≤ 4.05, > 4.05	1.062(0.626–1.800)	0.825		
Creatinine(umol/L): ≤ 85.8, > 85.8	1.908(1.137–3.202)	0.014	1.680(0.975–2.894)	0.061
Calcium(mmol/L): ≤ 2.35, > 2.35	1.370(0.790–2.376)	0.263		
Number of MetS components:0(Ref)	1.0(Ref)	Ref	1.0(Ref)	Ref
1	2.978(1.488–5.960)	0.002	4.487(0.552–36.454)	0.160
2	1.241(0.742–2.075)	0.410	11.738(1.532–89.934)	0.018
3–4	3.146(1.840–5.380)	0.001	21.967(2.851–169.263)	0.003

*Ref* reference, *OR* odds ratio, *CI* confidence interval

Eighty-five patients were MetS( +) and 225 were MetS(-). The operative time, haemoglobin deficit and stone-free rate were comparable in the two groups. A higher overall complication rate was found in the MetS( +) group (29 (34.12%) vs. 33 (14.67%), *P* < 0.001) (Table 4). Postoperative complication rates were high in the MetS( +) group (34.12% vs. 14.67%, *P* < 0.001), especially in patients with grade 1 fever (> 38 °C) and grade 2. The incidence of grade 3 or higher complications were comparable between the groups (Table 5).

**Table 4:** The effect of metabolic syndrome on percutaneous nephrolithotomy for staghorn stone

Variable	MetS( +)(85)	MetS(-)(225)	*P* value
Mean (SD)			
Operative time, min	93.09(31.54)	95.89(27.33)	0.442
Haemoglobin deficit, g/L	7.65(8.77)	8.44(7.87)	0.444
N (%)			
Complications	29(34.12)	33(14.67)	< 0.001
SFR	52(61.18)	124(55.11)	0.338

*MetS* Metabolic syndrome, *SD* standard deviation, *N* number, *SFR* stone free rate

**Table 5:** Postoperative complications

Modified Clavien system	MetS( +)(85)	MetS(-)(225)	*P* value
Complication rate, % (n)	29(34.12)	33(14.67)	< 0.001
Grade 1, n			
Fever (> 38 °C)	14	14	0.005
Grade 2, n	10	9	0.011
Blood transfusion	2	1	
Heart failure	2	-	
Nonoperative of perirenal hematoma	6	8	
Grade 3a, n			
Bleeding needs bladder irrigation	1	3	0.913
Grade 3b, n	2	3	0.526
Renal hemorrhage requiring angioembolization	2	-	
Nephrectomy due to hemorrhage	-	2	
Bladder rupture due to bleeding	-	1	
Grade 4a, n			
Bacteremia lead to shock	2	4	0.744
Grade 4b, n			
Multiple organ failure	-	-	
Grade 5, n			
Death due to septic shock	-	-	
≥ Grade 3 severity complication rate, n	5	10	0.600

*MetS* metabolic syndrome, *n* number

Furthermore, univariable and multivariable regression analyses were adopted to analyse the individual role of every component of MetS, the number of MetS components, and the MetS in Table 6 to assess their impact on complications. Univariable analysis showed that hyperlipidaemia and MetS were associated with a higher risk of complications. Multivariable logistic regression found that hyperlipidaemia (OR = 1.991, 95% CI 1.020–3.888, *P* = 0.044) and MetS (OR = 2.427, 95% CI 1.316–4.477, *P* = 0.005) were independent risk factors for complications. We found that the OR values increased as the number of MetS features increased by grade (0–4) but did not reach statistical significance.

**Table 6:** Analysis of the relationship between overall complications and metabolic syndrome components

Variable	Univariable analysis			Multivariable analysis		
	P	OR	95%CI	P	OR	95%CI
Number of MetS components				-	-	-
0(Ref)	-	-	-			
1	0.977	0.985	0.345–2.810			
2	0.126	0.427	0.144–1.270			
3	0.253	1.826	0.650–5.129			
4	0.077	3.200	0.881–11.627			
BMI	0.09	1.626	0.926–2.855	-	-	-
Hyperlipidemia	0.003	2.623	1.393–4.938	0.044	1.991	1.020–3.888
Hypertension	0.078	1.654	0.945–2.895	-	-	-
Diabetes mellitus	0.133	1.620	0.863–3.042	-	-	-
MetS	< 0.001	3.013	1.685–5.386	0.005	2.427	1.316–4.477

*MetS* metabolic syndrome, *Ref* reference, *BMI* body mass index, *OR* odds ratio, *CI* confidence interval

## Discussion

Staghorn calculus is one type of complex renal stone, and most studies have focused on therapy. Fewer studies have investigated the metabolism of blood and urine or MetS. To our knowledge, this is the first study focused on the incidence of MetS in patients with unilateral staghorn renal stones and patients with bilateral staghorn renal stones.

An updated cross-sectional study showed that the prevalence of MetS in China was 14.39% in adults over 18 years old [6]. However, the proportion of MetS in the United States increased to 34.1% from 1999 to 2006 [19], indicating its significance as a public health concern. Few studies have investigated the incidence of MetS in patients with unilateral renal stones and in patients with bilateral renal stones, especially staghorn calculi. A former study developed by Fan et al. [15] compared the metabolic differences between unilateral and bilateral renal stones. They found that the incidence of unilateral renal stones was 4.9%, and that of bilateral renal stones was 0.7%. Patients with bilateral renal stones (29.7%) had the highest incidence of chronic kidney disease, followed by unilateral renal stones (19.2%) and non-stone (11.0%). A study [14] compared the incidence of metabolic disorders between patients with unilateral and bilateral staghorn renal stones found cystine urine and blood urea nitrogen were higher in patients with bilateral renal stones. Our study found that there were no significant differences in serum calcium, magnesium, potassium, uric acid, or creatinine between patients with unilateral and those with bilateral staghorn renal stones. This may be due to the higher proportion of partial staghorn renal stones because a complete staghorn calculus may cause obstruction of the renal pelvis and calices, which may lead to serious hydronephrosis and result in significant changes in metabolic indicators.

Our study first reported the incidence of MetS in patients with unilateral staghorn renal stones and in patients with bilateral staghorn renal stones and then reported that patients with bilateral staghorn renal stones had a higher proportions of BMI, hypertension, diabetes, hyperlipidaemia, and MetS than those with unilateral staghorn renal stones. A previous study showed that urolithiasis was an independent risk factor for an increased risk of metabolic syndrome [6]. To examine the association between metabolic syndrome and the risk of kidney stone development, A team conducted a large-scale research with 121,579 participants [20], they found that the probability of kidney stones occurring in patients with metabolic syndrome was 1.79-fold than the patients with no metabolic syndrome. Our team’s previous research found that the overall incidence rate of metabolic syndrome in urinary stones was 22.6% [12]. This study found that the incidence rate of metabolic syndrome of unilateral staghorn calculus was 20.8%, but for patients with bilateral staghorn calculi, the incidence of metabolic syndrome reaches 45.24%. And we found that as the number of metabolic syndrome components increases, the risk of bilateral staghorn calculi increases, when the number of MetS components increases from 0 to 3–4(Mets( +)), the likelihood of developing bilateral staghorn calculi increases by 21.967 times. Therefore, we should pay more attention to patients with bilateral staghorn renal stones and conduct metabolic assessments.

With regard to the risk factors for complications after PCNL, most studies discussed the size of the track [21], experience of the operator [22], preoperative stone-related examination [23] and so on. Previous studies have rarely focused on MetS. Tefekli and colleagues [24] conducted a retrospective study to discuss the effect of MetS and its components on the incidence of complications after PCNL and found that patients with hypertension, diabetes, and MetS were 2.5, 2.7, and 2.45 times more likely to experience major complications than patients lacking the above factors. Johans and colleagues [16] first assessed the relationship between MetS and myocardial infarction after PCNL and found that the presence of three-four components of MetS increased the odds of postoperative myocardial infarction. Xu and colleagues [17] first addressed the effect of the number of MetS components on the outcome of PCNL in staghorn renal stones patients. The number of MetS components was calculated on a scale of zero to five. The results showed that the number of MetS components as the central predictor was suitable for assessing the severity of MetS and the risk of PCNL. Having three, four, or even five MetS components increase the risk for PCNL. In this study, postoperative complication rates were high in the MetS( +) group (34.12% vs. 14.67%, *P* < 0.001), especially grade 1 (fever > 38 °C) and grade 2 complications. This is similar to previous research results. We found that there was no difference in the incidence of severe postoperative complications between the two groups of patients, possibly due to the relatively small number of grade 3 and higher postoperative complications reflected in our data. Tract dilatation with a 16F dilator was most popular, and a smaller tract was associated with reduced the incidences of bleeding and blood transfusion complications. There is a great difference in the surface area of renal parenchymal violation between mini-PCNL and standard-PCNL [25]. Previous research has shown that low high-density lipoprotein and high triglycerides are associated with kidney stone formation, which may be explained by systemic inflammation and oxidative stress [26, 27]. Our study showed that the difference in the incidence of complications between Mets( +) and MetS(-) lies in grade 1 and grade 2 and is mainly focused on postoperative inflammatory reactions and haemorrhagic diseases, which may further explain why hyperlipidaemia can exacerbate postoperative complications. MetS was an independent risk factor for complications of staghorn renal stones, which was confirmed by previous research [17].

The MetS components were divided into 0–4 components (obesity, hypertension, diabetes mellitus and hyperlipidaemia) as MetS is diagnosed by assessing the above four indicators. Most papers have found that the risk of postoperative complications increases as the number of MetS components increase [16, 17]. We found that the OR values increased as the number of MetS features increased by grade but did not reach statistical significance. For patients with metabolic syndrome, especially hypertension and diabetes, we tried our best to control the relevant indicators within a reasonable range before surgery. Therefore, the number of MetS may not be the primary factor influencing the incidence of complications after PCNL for staghorn calculi.

Our study found that the operative time, haemoglobin deficit and stone-free rate were comparable between the MetS( +) and MetS(-) groups. This was consistent with the research results reported by Akman et al. [28]. The main factors influencing operative time, haemoglobin deficit and stone-free rate are most related to hydronephrosis, stone size, and puncture tract, not MetS.

Patients with SC stones should undergo routine metabolic assessment before surgery, especially for bilateral SC. For patients with MetS, it is necessary to actively control the relevant components of metabolic syndrome to achieve the desired level before surgery to reduce the occurrence of postoperative complications. Postoperative complication rates were high in the MetS( +) group, especially in patients with grade 1 fever (> 38℃) and grade 2 (blood transfusion, heart failure, nonoperative of perirenal hematoma), so active anti infection treatment during the perioperative period can reduce postoperative infections. Pay attention to the drainage tube unobstructed, detect the bleeding, and handle them early to avoid serious complications occurrence.

The limitation of our study is that it was a retrospective analysis, we cannot conclude the causal relationship between MetS and SC, which will require prospective studies to determine. Due to limitations of examination equipment in earlier years, no stone component analysis was conducted, resulting in us not being able to conduct subgroup analysis on the advantages and disadvantages of the two methods for treating SC with different components. Failure to collect 24-h urine from patients for analysis, which is crucial for an accurate analysis of metabolic conditions and the identification of factors that may contribute to stone formation. Because of race differences, the diagnostic criteria for MetS differs between China and foreign countries, the specific thresholds and measurements used to define MetS can vary based on genetic, environmental, and lifestyle factors that are unique to different populations. As a result, when comparing the prevalence and characteristics of MetS between Chinese populations and those in other countries, discrepancies may arise due to these differing diagnostic standards. 

Based on the findings of this study, several specific future research directions are recommended to encourage further exploration in this area. Studies should aim to establish standardized protocols for the collection and analysis of 24-h urine samples. This will ensure more consistent and reliable data, aiding in the identification of metabolic factors contributing to SC formation; Future research should focus on developing stone component analysis, and form a treatment plan for SC based on the composition of stones. By addressing these research directions, the medical community can enhance its understanding of SC and MetS, ultimately leading to improved patient outcomes from PCNL. 

## Conclusion

MetS is correlated with the formation of bilateral staghorn renal stones and is the main predictor for complications of PCNL of staghorn renal stones especially for low-grade complications (I-II). Attention should be given to the evaluation of MetS in patients with staghorn calculi.

## Data Availability

All data and material generated or used during the study are included in the submitted article.

## Abbreviations

MetS: Metabolic syndrome
PCNL: Percutaneous nephrolithotomy
OR: Odds ratio
BMI: Body mass index
HDL-C: High-density lipoprotein cholesterol
SFR: Stone free rate
ROC: Receiver Operating Characteristic
SC: Staghorn calculi
N/n: Number
CI: Confidence interval
TG: Triglycerides
SD: Standard deviation
R: Reference
Fr: French
CT: Computed tomography
HU: Hounsefield units

## References

[1] Scales CJ, Smith AC, Hanley JM, Saigal CS (2002). Prevalence of kidney stones in the United States. Eur Urol.

[2] Zeng G, Mai Z, Xia S, Wang Z, Zhang K, Wang L (2017). Prevalence of kidney stones in China: an ultrasonography based cross-sectional study. BJU Int.

[3] Preminger GM, Assimos DG, Lingeman JE, Nakada SY, Pearle MS, Wolf JS (2005). Chapter 1: AUA guideline on management of staghorn calculi: diagnosis and treatment recommendations. J Urol..

[4] Türk C, Petřík A, Sarica K, Seitz C, Skolarikos A, Straub M (2016). EAU guidelines on interventional treatment for urolithiasis. Eur Urol.

[5] Michel MS, Trojan L, Rassweiler JJ (2007). Complications in percutaneous nephrolithotomy. Eur Urol.

[6] Lan Y, Mai Z, Zhou S, Liu Y, Li S, Zhao Z (2018). Prevalence of metabolic syndrome in China: an up-dated cross-sectional study. PLoS ONE.

[7] Otto BJ, Bozorgmehri S, Kuo J, Canales M, Bird VG, Canales B (2017). Age, body mass index, and gender predict 24-hour urine parameters in recurrent idiopathic calcium oxalate stone formers. J Endourol.

[8] Hartman C, Friedlander JI, Moreira DM, Elsamra SE, Smith AD, Okeke Z (2015). Differences in 24-h urine composition between nephrolithiasis patients with and without diabetes mellitus. BJU Int.

[9] Abu-Ghanem Y, Shvero A, Kleinmann N, Winkler HZ, Zilberman DE (2018). 24-h urine metabolic profile: is it necessary in all kidney stone formers?. Int Urol Nephrol.

[10] Hartman C, Friedlander JI, Moreira DM, Leavitt DA, Hoenig DM, Smith AD (2015). Does hypertension impact 24-hour urine parameters in patients with nephrolithiasis?. Urology.

[11] Rahman IA, Nusaly IF, Syahrir S, Nusaly H, Mansyur MA (2021). Association between metabolic syndrome components and the risk of developing nephrolithiasis: a systematic review and bayesian meta-analysis. F1000Res..

[12] Fu Q, Xie L, Diao C, Aizezi X, Liu X, Liu C (2022). The impacts of metabolic syndrome on the risk of severe urolithiasis. Urolithiasis.

[13] Amaro CR, Goldberg J, Agostinho AD, Damasio P, Kawano PR, Fugita OE (2009). Metabolic investigation of patients with staghorn calculus: is it necessary?. Int Braz J Urol.

[14] Mohammadi SM, Jafarpisheh A, Ghoreifi A (2019). Evaluation and comparison of metabolic disorders between patients with unilateral and bilateral staghorn renal stones. Urol J.

[15] Fan X, Ye W, Ma J, Wang L, Heng W, Zhou Y (2022). Metabolic differences between unilateral and bilateral renal stones and their association with markers of kidney injury. J Urol.

[16] Johans CE, Bajic P, Kirshenbaum E, Blackwell RH, Kothari AN, Kuo PC (2018). Metabolic syndrome increases risk of postoperative myocardial infarction following percutaneous nephrolithotomy. J Endourol.

[17] Xu P, Wang J (2019). Number of metabolic syndrome components is the central predictor of the impact of metabolic syndrome on outcome of percutaneous nephrolithotomy in staghorn nephrolithiasis. J Endourol.

[18] Dindo D, Demartines N, Clavien PA (2004). Clavien, classification of surgical complications: a new proposal with evaluation in a cohort of 6336 patients and results of a survey. Ann Surg.

[19] Mozumdar A, Liguori G (2011). Persistent increase of prevalence of metabolic syndrome among U.S. adults: NHANES III to NHANES 1999–2006. Diabetes Care..

[20] Chang CW, Ke HL, Lee JI, Lee YC, Jhan JH, Wang HS (2021). Metabolic syndrome increases the risk of kidney stone disease: a cross-sectional and longitudinal cohort study. J Pers Med.

[21] Feng D, Hu X, Tang Y, Han P, Wei X (2020). The efficacy and safety of miniaturized percutaneous nephrolithotomy versus standard percutaneous nephrolithotomy: a systematic review and meta-analysis of randomized controlled trials. Investig Clin Urol.

[22] Akman T, Binbay M, Kezer C, Yuruk E, Tekinarslan E, Ozgor F (2012). Factors affecting kidney function and stone recurrence rate after percutaneous nephrolithotomy for staghorn calculi: outcomes of a long-term followup. J Urol.

[23] Grosso AA, Sessa F, Campi R, Viola L, Polverino P, Crisci A (2021). Intraoperative and postoperative surgical complications after ureteroscopy, retrograde intrarenal surgery, and percutaneous nephrolithotomy: a systematic review. Minerva Urol Nephrol.

[24] Tefekli A, Kurtoglu H, Tepeler K, Karadag MA, Kandirali E, Sari E (2008). Does the metabolic syndrome or its components affect the outcome of percutaneous nephrolithotomy?. J Endourol.

[25] Khadgi S, El-Nahas AR, El-Shazly M, Al-Terki A (2021). Comparison of standard- and mini-percutaneous nephrolithotomy for staghorn stones. Arab J Urol.

[26] Torricelli FC, De SK, Gebreselassie S, Li I, Sarkissian C, Monga M (2014). Dyslipidemia and kidney stone risk. J Urol.

[27] Gorbachinsky I, Akpinar H, Assimos DG (2010). Metabolic syndrome and urologic diseases. Rev Urol.

[28] Akman T, Binbay M, Erbin A, Tepeler A, Sari E, Kucuktopcu O (2012). The impact of metabolic syndrome on long-term outcomes of percutaneous nephrolithotomy (PCNL). BJU Int.

